# Associations of brain–natriuretic peptide, high–sensitive troponin T, and high–sensitive C–reactive protein with outcomes in severe aortic stenosis

**DOI:** 10.1371/journal.pone.0179304

**Published:** 2017-06-12

**Authors:** Andreas Auensen, Amjad Iqbal Hussain, Ragnhild Sørum Falk, Marte Meyer Walle-Hansen, Jorun Bye, Kjell Ingar Pettersen, Pål Aukrust, Thor Ueland, Lars Lysgaard Gullestad

**Affiliations:** 1 Department of Cardiology, Oslo University Hospital, Rikshospitalet, Oslo, Norway; 2 Centre for Heart Failure Research, Faculty of Medicine, University of Oslo, Oslo, Norway; 3 Faculty of Medicine, University of Oslo, Oslo, Norway; 4 Oslo Centre for Biostatistics and Epidemiology, Research Support Services, Oslo University Hospital, Ullevål, Oslo, Norway; 5 Research Institute of Internal Medicine, Oslo University Hospital Rikshospitalet, Oslo, Norway; 6 Section of Clinical Immunology and Infectious Diseases, Oslo University Hospital, Rikshospitalet, Oslo, Norway; 7 K.G. Jebsen Thrombosis Research and Expertise Center, University of Tromsø, Tromsø, Norway; Osaka University Graduate School of Medicine, JAPAN

## Abstract

**Objectives:**

Among patients with severe aortic stenosis (AS), we investigated the associations of N–terminal pro–natriuretic peptide (NT–proBNP), high–sensitive troponin T (hsTnT), and high–sensitive C–reactive protein (hs–CRP) with 3–year mortality and major adverse cardiovascular events (MACE) during 1 year.

**Methods:**

This observational cohort study prospectively enrolled 442 patients with severe AS who were referred for evaluation of possible valve replacement. Clinical data was recorded before the decision of whether to operate was made. We studied the prognostic value of assessing biomarkers by serum levels, and tested associations of NT–proBNP, hsTnT, and hs–CRP with clinical outcomes (3–year all–cause mortality and risk of MACE in the year following study inclusion) using adjusted multivariable analysis.

**Results:**

Elevated serum levels of these biomarkers at baseline evaluation were associated with increased all–cause 3–year mortality regardless of treatment assignment. Adjusted analysis showed that none of the studied biomarkers (NT–proBNP, hsTnT or hs–CRP) or any other covariates were associated with 3–year all–cause mortality following surgical aortic valve replacement (SAVR). However, adjusted analyses showed that hsTnT (HR, 1.51; 95% CI, 1.11–2.05; P = 0.008) and left ventricular ejection fraction (HR 0.97; 95% CI 0.94–0.97, P = 0.043) was associated with MACE for operated patients.

**Conclusions:**

Whereas NT–proBNP, hsTnT and hs–CRP had no independently prognostic value in relation to all–cause mortality following SAVR, hsTnT was independently associated with MACE following operation. The use of these clinically available biomarkers, in particular hsTnT, should be clarified in larger studies.

## Introduction

Aortic valve stenosis (AS) is the most common valvular heart disease in the developed world [1, 2]. Since the prevalence of AS increases with age, it is expected that the number of patients requiring treatment will rise as the population ages [3]. Without treatment, patients with severe symptomatic AS face a dismal prognosis, showing up to 50% mortality over 2 years [4, 5]. In patients with severe AS and low–to–moderate surgical risk, surgical aortic valve replacement (SAVR) is the gold standard of treatment. Among these patients, symptom development marks the transition from watchful waiting to recommending with aortic valve replacement [6].

The evaluation of subjective symptoms in AS is challenging since the majority of individuals scheduled for SAVR are elderly and have multiple comorbidities. The use of biomarkers could improve risk stratification in patients with AS, and aid in clinical decision–making by providing prognostic information. Recent studies using N–terminal pro–brain natriuretic peptide (NT–proBNP) and high–sensitive troponin T (hsTnT) have demonstrated the value of biomarkers for evaluating patients with AS [7–9]. However, except for in a small study by our group [10], the combined use of these biomarkers along with high–sensitive C–reactive protein (hs–CRP) in patients with AS has not been reported.

The clinically available NT–proBNP, hsTnT, and hs–CRP are robust standard biomarkers of myocardial stress, myocardial damage, and inflammation, respectively. Here, we hypothesized that these biomarkers, and their serum levels relative to normal, would be useful prognostic factors of mortality when assessing patients referred to a tertiary centre for evaluation of potential valve replacement. As possible markers of various aspects of the pathophysiological disease process in AS, we further hypothesized that these markers would be associated with the risk of major adverse cardiovascular events (MACE) in the year following SAVR.

## Materials and methods

### Study design and patient population

Our observational cohort included patients with severe AS who were referred to our tertiary centre (Oslo University Hospital Rikshospitalet, Norway) to be evaluated for possible aortic valve replacement (AVR) between May 2010 and March 2013. Inclusion criteria were age > 18 years, severe AS and the ability to read and write in Norwegian. Patients were excluded if they did not have severe AS, were unwilling to participate, or had undergone previous AVR. We also excluded subjects scheduled for transcatheter aortic valve implantation (TAVI) due to the limited number of transcatheter interventions performed during this period.

Severe AS was defined in accordance with current guidelines [6]. In cases of a low–flow, low–gradient state, with either a preserved or reduced left ventricular ejection fraction (LVEF), patients were further evaluated using low–dose dobutamine stress and/or transesophageal echocardiography.

Being an observational study, the heart team was blinded to additional study data. We reviewed the medical records for all patients during the year following study inclusion. Data regarding adverse events and hospitalizations were collected from the patients’ operating and local hospitals. The study protocol was approved by the regional committee for ethics in medicine, included approval from local hospitals, complied with the Declaration of Helsinki, and was registered at Clinicaltrials.gov (NCT01794832). All patients signed a written informed consent before study participation.

### Clinical data collection at baseline

Patients underwent clinical and physical examinations at baseline that included resting blood pressure evaluation, standard resting 12–lead electrocardiogram (ECG), peripheral blood sampling, and transthoracic echocardiography. Echocardiography was performed using Vivid 7 or E9 ultrasound scanners (GE Vingmed Ultrasound, Horten, Norway). Functional status and/or symptoms of exertional dyspnea or angina were evaluated based on New York Heart Association functional class (NYHA). Aortic valve stenosis severity was assessed by continuous wave Doppler through multiple acoustic windows to obtain the maximal jet velocity. We measured maximal instantaneous and mean pressure gradients across the aortic valve using the time velocity integral, and estimated the aortic valve area using the continuity equation. Left ventricular ejection fraction (LVEF) was calculated using the modified Simpson biplane method.

### Biochemistry and blood sampling

Peripheral venous blood was drawn into pyrogen–free tubes containing EDTA as an anticoagulant. These tubes were immediately immersed in melting ice. Within 30 minutes of collection, the tubes were centrifuged at 2000 g for 20 minutes to obtain platelet–poor plasma. All samples were stored at −80°C until analysis. We determined the plasma concentrations of NT–proBNP using an electrochemiluminescence immunoassay on a Modular platform (Roche Diagnostica, Basel, Switzerland). The coefficient of variation (CV) was < 4%, and the lower detection limit was 5.1 ng/L. At the core lab of our hospital, the upper limits of the normal ranges were as follows: < 169 ng/L for women, 18−49 years old; < 296 ng/Lfor women, 50−69 years old; < 761 ng/L for women, ≥70 years old; < 85 ng/L for men, 18−49 years old; < 254 ng/L for men, 50−69 years old; and < 507 ng/L for men, ≥70 years old. We assessed hs−CRP levels using a particle–enhanced, high−sensitive immunoturbidimetric assay (hs−CRP, Tina−Quant CRP Gen.3), which has a minimal detectable concentration of 0.6 mg/L, and a total imprecision of ≤ 7.5% for C–reactive protein <5 mg/L. The normal range is < 4 mg/L. High−sensitive TnT was measured by electrochemiluminescence immunoassay (hsTnT, Elecsys Troponin T high sensitive, Roche Diagnostics), which has a blank limit of 3 ng/L. The lowest concentration with a CV of ≤10% was 13 ng/L. Values in the range of 12−14 ng/L are within the 99th percentile in a healthy control cohort (Roche Diagnostics GmbH, D−68298 Mannheim).

### Mortality and major adverse cardiovascular events (MACE)

For all patients, we retrieved complete 3–year all–cause mortality data from The Norwegian National Cause of Death Registry and the causes of death was obtained from the Norwegian Cause of Death Registry. We also reviewed the patients’ medical records from their operating–and local hospitals to acquire data regarding all adverse clinical events during the year following study inclusion. The composite endpoint of MACE comprised the time from baseline to diagnosis of transient ischemic attack (TIA), stroke, myocardial infarction (MI), or all–cause death. To ensure completeness of data, we reviewed the official clinical coding system for diagnosis and procedures used in Norway (International Statistical Classification of Diseases and Related Health Problems; ICD–10), and read the journal texts of each medical record.

### Statistical analyses

Data analyses were performed using STATA version 14 (StataCorp. 2015. Stata Statistical Software: Release 14. College Station, TX: StataCorp LP). Descriptive statistics are provided as frequencies and proportions for categorical variables, and as mean and standard deviation (SD) or as median and interquartile range (IQR) as appropriate for continuous variables. Means and proportions were compared using χ² tests for categorical variables or *t*–tests or the Mann–Whitney test for continuous variables. For the analyses, patients were categorized by the intention–to–treat principle based on the decision taken by the heart team at the time of deciding whether to operate or not.

We performed two sets of follow–up analyses: one including MACE, and the other focused on all–cause mortality. In the former analysis, patients were followed from the date of inclusion (operation day for operated, or day of outpatient evaluation for unoperated patients) to the date of MACE. Patients were censored at one year after inclusion. In the analysis focused on all–cause mortality, patients were followed from date of inclusion to their date of death or censored after three years. We obtained complete 3–year mortality data for all included patients.

To evaluate associations between biomarkers and outcomes, we performed Cox–regression analysis with adjustment for the following potential confounding factors: age at inclusion, gender, estimated glomerular filtration rate (eGFR), NYHA class and LVEF. The aforementioned covariates were included in the multivariate model regardless of their *P* values, whereas NT–proBNP, hsTnT, and hs–CRP were selected using a forward stepwise approach. Biomarkers showing significance at a *P* value of < 0.1 in univariate analysis were introduced in the stepwise selection, while only the statistically significant biomarkers (*P* < 0.05) remained in the model. Variables with skewed distributions were log–transformed and then presented as Z–scores. To assess strength of the association between each variable and the outcome, we calculated the hazard ratio (HR) and 95% confidence interval (CI). Graphical analysis verified that the assumptions of proportional hazards were adequately met.

## Results

### Clinical characteristics and association with biomarkers

**Fig 1:**
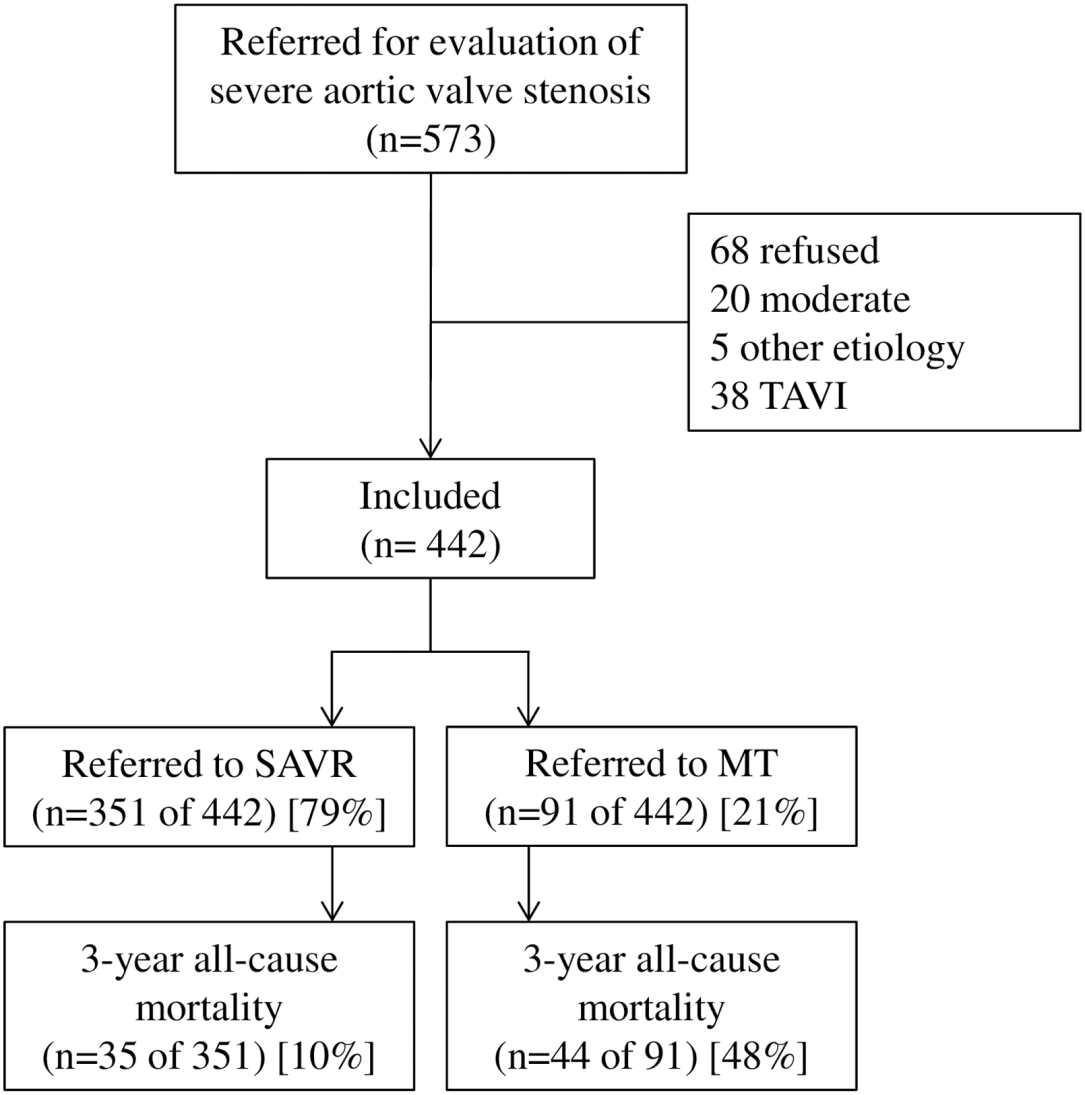
Study flow. TAVI, transcatheter aortic valve implantation; SAVR, surgical aortic valve replacement; MT, medical treatment.

Of the 573 consecutively registered eligible patients, 68 declined participation, 5 received other diagnoses, 20 had moderate AS, and 38 were excluded following referral for TAVI (Fig 1). Among the 442 included patients, 91 (20.6%) did not undergo surgery, due to either lack of symptoms (n = 34), a high risk–benefit ratio (n = 37), or refusal (n = 20). The remaining 351 patients (79.4%) had low–moderate surgical risk (Euro(II)score < 8 in 94.6% of included patients) and underwent SAVR at Oslo University Hospital. Three patients died after referral while awaiting SAVR. Among the operated patients, 279 (79.5%) received bioprosthetic valves, and 103 (29.3%) underwent concomitant CABG. For operated patients, the median stay before transfer was 3 days (IQR, 3–4), and the median duration of continued postoperative recovery in a local hospital was 6 days (IQR, 4–11).

Table 1 presents the baseline characteristics of operated and unoperated patients. Operated patients were younger; had higher BMI; showed lower frequencies of atrial fibrillation and DM; less often used warfarin, had better renal function (lower se–creatinine and higher eGFR) and a lower Euro(II)score. Furthermore, operated patients had different NYHA–class distribution; higher cardiac output, aortic valve peak velocity and aortic mean gradient; lower NT–proBNP–levels and higher hs–TnT. Supporting Information (S1–S3 Tables) display the patients’ clinical characteristics according to tertiles of NT–proBNP, hsTnT, and hs–CRP.

**Table 1:** Baseline characteristics.

Variables	All patients	Surgical AVR	Unoperated	
	n = 442	n = 351	n = 91	p–value
**Demography**				
Mean age, years	74 ± 11	73 ± 10	81 ± 9	<0.0001
Male sex–n (%)	249 (56)	205 (58)	44 (48)	0.085
Body mass index, kg/m²	26 ± 5	26 ± 4	25 ± 5	<0.0001
**Medical history–**n (%)				
Hypertension	202 (46)	162 (46)	40 (44)	0.708
Atrial fibrillation, all types	94 (21)	65 (19)	29 (32)	<0.006
Diabetes mellitus type I and II	48 (11)	31 (9)	17 (19)	<0.007
Coronary artery disease	131 (30)	106 (30)	25 (27)	0.612
**Medication–**n (%)				
Betablocker	206 (47)	157 (45)	49 (54)	0.120
ACEi/ARB	178 (40)	135 (38)	43 (47)	0.262
Warfarin	90 (20)	61 (17)	29 (32)	0.002
Platelet inhibitor	231 (52)	190 (54)	41 (45)	0.122
**Surgical risk score**				
Euro(II)SCORE, median ± IQR	2 (1, 4)	2 (1, 4)	4 (2, 8)	<0.0001
**NYHA classification–**n (%)				<0.0001
Class I	48 (11)	21 (6)	27 (30)	
Class II	205 (46)	176 (50)	29 (32)	
Class III/IV	189 (43)	154 (44)	35 (38)	
**Hemodynamics**				
LVEF, %	56.2 ± 9.2	56.4 ± 8.6	55.1 ± 11.2	0.227
Cardiac output, l/min	4.9 ± 1.2	4.9 ± 1.2	4.6 ± 1.3	0.034
Aortic peak velocity, m/s	4.7 ± 0.8	4.7 ± 0.8	4.5 ± 0.7	0.021
Aortic mean gradient, mmHg	55 ± 18	56 ± 18	51 ± 18	0.013
Aortic valve area, cm²	0.7 ± 0.2	0.7 ± 0.2	0.7 ± 0.2	0.253
**Biochemistry**				
Creatinine, μmol/L	87 ± 29	85 ± 28	93 ± 31	0.027
eGFR, ml/min	74 ± 33	78 ± 32	58 ± 29	<0.0001
NT–pro–BNP, ng/L median (IQR)	770 (279, 1979)	600 (254, 1505)	1624 (491, 3400)	<0.0001
hs–TnT, ng/mL median (IQR)	14 (10, 25)	12 (10, 22)	19 (12, 38)	<0.0001
hs–CRP, mg/l	5.1 ± 9	4.7 ± 8	6.6 ± 12	0.085

Plus–minus values are means ±SD. P–values for comparison between operated and unoperated patients. IQR, interquartile range; ACEi, angiotensin converting enzyme inhibitor; ARB, angiotensin receptor blocker; Euro(II)SCORE, European System for Cardiac Operative Risk Evaluation; NYHA, New York Heart Association; LVEF, left ventricular ejection fraction; eGFR, estimated glomerular filtration rate (Cockcroft–Gault formula); NT–proBNP, N–terminal pro brain natriuretic peptide; hs–TnT, high–sensitive troponin T hs–CRP, high–sensitive C–reactive protein.

### Mortality and MACE

Three–year all–cause mortality was 10.0% (n = 35) among patients who underwent SAVR, and 48.4% (n = 44) among patients referred for continued medical treatment. In the total population, the number of cardiac deaths were 47 of 79 (59.5%) at 3 years from inclusion, whereas 16 of 35 (45.7%) of operated patients and 31 of 44 (70.5%) of unoperated patients died cardiac deaths.

Table 2 displays the causes of MACE for operated and unoperated patients. After surgery, 43 patients (12.3%) experienced at least one MACE during the following year (incidence rate, IR 13.5 per 100 patient years). The distribution of reasons (based on the relative proportion of total events defined as MACE) was as follows: 27.9% all–cause mortality, 16.3% TIA, 46.5% stroke, and 9.3% MI. For patients referred to medical treatment, 21 patients (23.1%) experienced MACE (IR, 26.3) during the following year. The distribution of reasons were 42.8% all–cause mortality, 0% TIA, 14.4% stroke, and 42.8% MI.

**Table 2:** Causes of major adverse cardiovascular events (MACE).

		All patients n = 442		SAVR n = 351		Unoperated n = 91	
		n (%)	Rel.%	n (%)	Rel.%	n (%)	Rel.%
MACE	All–cause death	21 (4.8)	33	12 (3.4)	27.9	9 (9.9)	42.8
	TIA	7 (1.6)	11	7 (2.0)	16.3	0 (0.0)	0
	Stroke	23 (5.2)	36	20 (5.7)	46.5	3 (3.3)	14.4
	MI	13 (2.9)	20	4 (1.1)	9.3	9 (9.9)	42.8
No MACE		376 (85.1)		308 (87.7)		70 (76.9)	

SAVR, surgical aortic valve replacement; TIA, transient ischemic attack, MI, myocardial infarction; MACE major adverse cardiovascular events. Rel.% refers to the proportion of events with total number of MACE in each group.

### The combination of elevated biomarkers as a prognostic factor of mortality

At initial presurgical evaluation before the decision of whether to operate was taken, 111 patients (25.3%) had levels of NT–proBNP, hsTnT and hs–CRP that were below the upper limit of the normal range (“all 3 normal”); 251 patients (57.3%) had measurements above the upper limit for at least one of the assessed biomarkers (“at least one elevated”); and 76 patients (17.4%) had measurements above the upper limit of the normal range for all three biomarkers (“all 3 elevated”). Four individuals were excluded from the analysis because one of the biomarkers was not measured. Among patients with all 3 normal, 8 of 111 (7.2%) died within 3 years. For patients with at least one of the biomarkers elevated, 3–year all–cause mortality was 42 of 251 (16.7%), and for patients with all 3 biomarkers elevated, 29 of 76 (38.2%) died within 3 years of inclusion. The risk of 3–year all–cause mortality was significantly different (Log–Rank Test, P < 0.001) in between the three aforementioned groups of biomarker levels.

**Fig 2:**
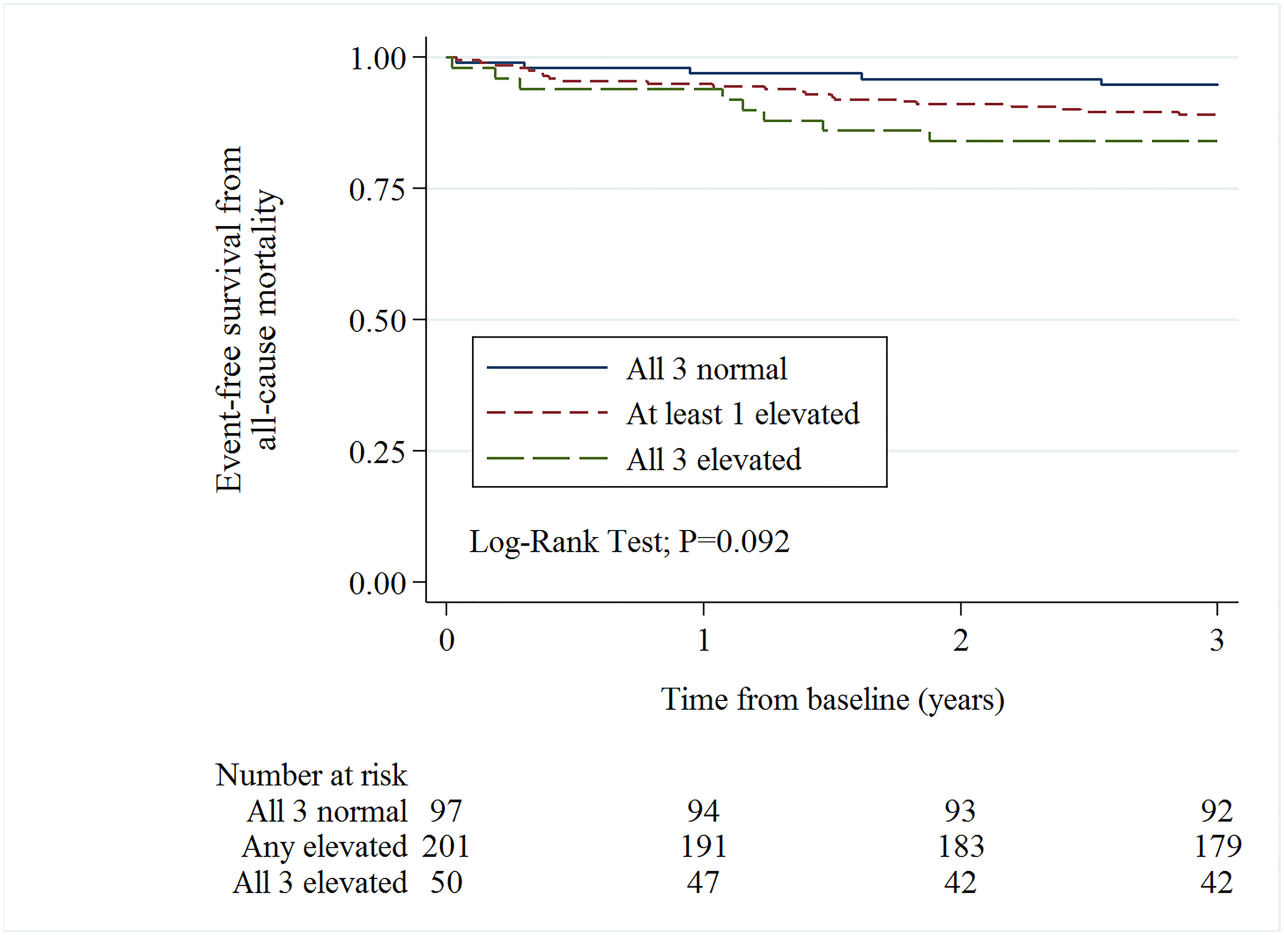
Kaplan Meier curves (3–year all–cause mortality) for operated patients with normal levels–, at least one elevated–or all three biomarkers elevated.

**Fig 3:**
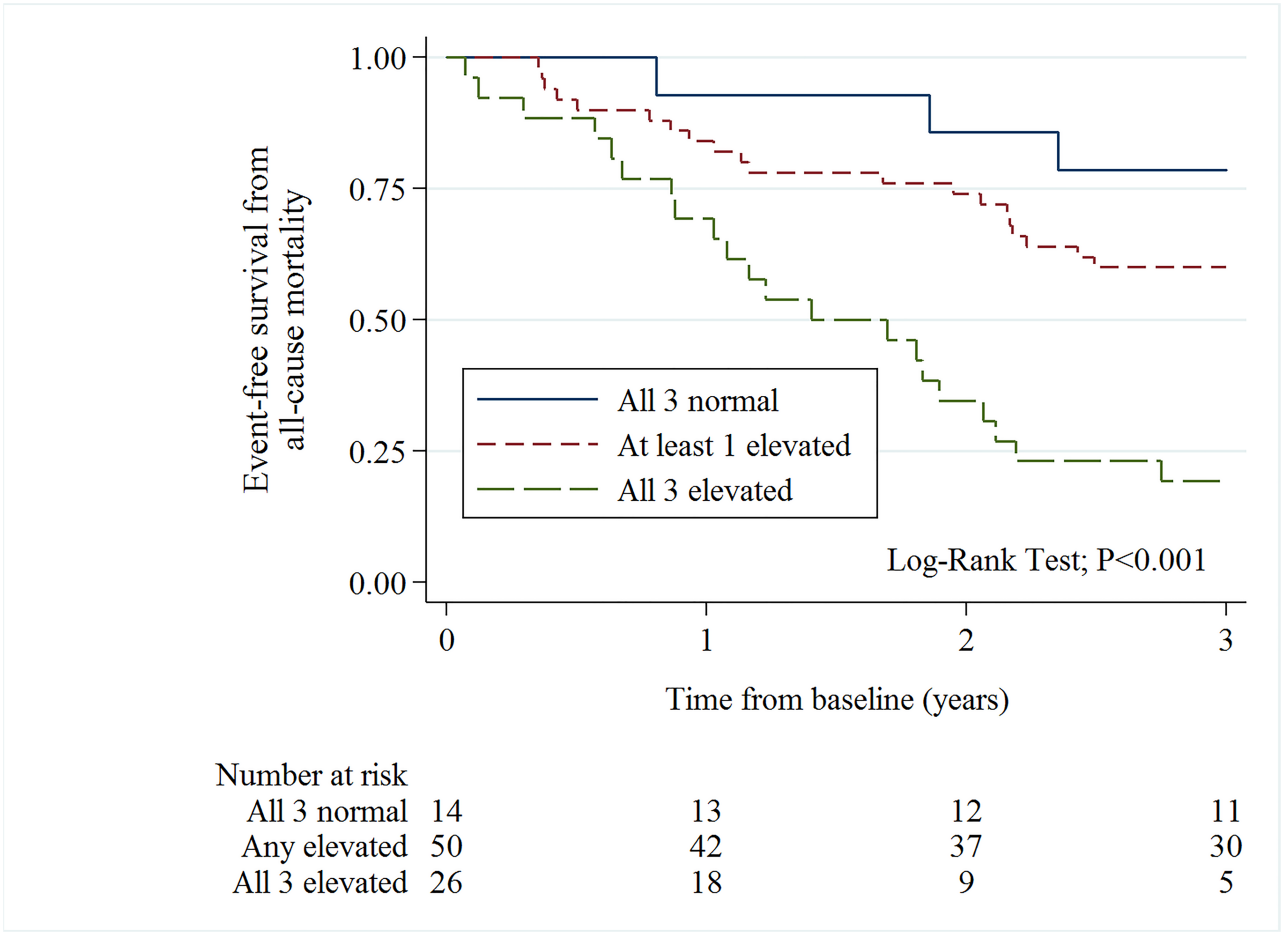
Kaplan Meier curves (3–year all–cause mortality) for unoperated patients with normal levels–, at least one elevated–or all three biomarkers elevated.

When analyzing the risk of death according to treatment decision, the all–cause mortality risk within 3 groups of biomarker levels (“all 3 normal”, “at least one elevated”, and “all 3 elevated”) was not statistically different in those patients referred to SAVR (Log–Rank Test, P = 0.092) (Fig 2). Among unoperated patients, there was a statistically significant difference among the groups of elevated biomarkers (Log–Rank Test, P<0.001) (Fig 3), but when comparing the difference between those who had all 3 normal (n = 14), vs. at least one elevated biomarker (n = 50), the difference was not statistically significant (Log–Rank Test; P = 0.206).

When analyzing the association between the clinical endpoint cardiac death with elevations of biomarkers (one, at least one, or all three of NT–proBNP, hsTnT and / or hs–CRP), our analyses demonstrate that the risk of 3–year cardiac–specific death was not statistically different between the “all 3 normal”, “at least one elevated”, and “all 3 elevated” groups in operated patients (LogRank–Test; P = 0.449) (S1 Fig). However, this analysis was restricted by relatively few events. On the other hand, among unoperated patients, the number of cardiac deaths was significantly different in the three groups (LogRank Test; P = 0.006) (S2 Fig). Interestingly, among operated patients, the 12 patients who died from cardiac causes died sooner after SAVR than those 18 patients who died from non–cardiac causes (LogRank–Test, P<0.001) (S3 Fig), while no such difference was observed among unoperated patients, despite that the number of cardiac deaths (n = 29) vs. non–cardiac deaths (n = 12) was larger in this group (LogRank–Test, P = 0.294) (S4 Fig).

### Association of biomarkers with all–cause mortality and MACE

In the Supporting Information, S4 Table displays univariate–and multivariable analyses for associations with all–cause 3–year mortality for operated patients. In a model including age, gender, eGFR, NYHA, LVEF and the three biomarkers at inclusion, no covariates were significantly associated with all–cause 3–year mortality. However, hsTnT (HR, 1.51; 95% CI, 1.11–2.05; P = 0.008) and LVEF (HR, 0.97; 95% CI, 0.94–0.97; P = 0.043) were significantly associated with MACE during one year in adjusted analysis including the same aforementioned covariates. Multivariate analysis in the unoperated group was restricted by the limited number of patients included.

## Discussion

In the present study, we investigated outcomes in a large cohort of patients with severe AS who were evaluated for possible AVR. By measuring baseline levels of NT–proBNP, hsTnT, and hs–CRP, we assessed the predictive value of these biomarkers for identifying patients at risk for adverse outcomes, including all–cause, cardiac–specific mortality and MACE. Whereas NT–proBNP, hsTnT and hs–CRP had no independently prognostic value in relation to mortality following SAVR, hsTnT was associated with MACE following surgery, also after adjustment for covariates. In the unoperated group, elevated levels of all three biomarkers was associated with increased all–cause–and cardiac–specific 3–year mortality when compared with having no elevated biomarker–levels.

Over the past decade, many studies have shown that natriuretic peptides are associated with mortality, extent of myocardial remodeling, transition from asymptomatic to symptomatic AS, and identification of patients at high risk of requiring SAVR [11–13]. Current guidelines recommend analysis of BNP as a marker of wall stress in the evaluation of patients with asymptomatic severe AS [6]. Clavel et al. [7] recently proposed the additional assessment of natriuretic peptide levels. They individualized the values for each patient through adjustment for age, gender, and measurement assays, and reported that this information improved risk prediction compared to BNP assessment alone among patients with moderate and severe AS. In the present study, findings suggest that NT–proBNP do not have a strong association with all–cause 3–year mortality. When performing multivariate analyses, we experienced that there was a statistically significant association between NT–proBNP and mortality before adding LVEF in the model, suggesting that these two clinical assessments may reflect similar pathways and thus not giving additional prognostic value when combined.

Interestingly, in this study hsTnT was the only biomarker that was significantly associated with MACE. Previous publications have demonstrated increased troponin levels in patients with AS [14, 15] and a recent study of 250 patients with stable aortic stenosis reported that >98% of patients had detectable high–sensitive troponin I levels (hsTnI), of which approximately 8% exceeded the threshold for MI diagnosis [8]. In the latter study the authors also showed that hsTnI was independently associated with LV mass and replacement fibrosis (late gadolinium enhancement on MRI), fueling interest in troponin as a valuable marker in AS. In the present study, elevated hsTnT was associated with MACE risk following SAVR. Although we cannot yet explain this pattern, it may partly reflect the fact that of the 43 events defined as MACE in operated patients, 62.5% were either a TIA or stroke, potentially indicating the activation of pathogenic pathways other than those related to all–cause mortality. In a study that followed over 400 patients for two years after MI, adjusted analysis revealed that hsTnI was associated with risk of cerebrovascular events (TIA/stroke) [16]. However, although raised hsTnT could be a marker of atherosclerotic disease, these findings do not fully explain the relationship between a cardiac–specific biomarker (e.g., hsTnT) and risk of cerebrovascular events. Nevertheless, our results suggest the potential usefulness of hsTnT as a marker of myocardial necrosis and replacement fibrosis in patients with symptomatic severe AS and as a risk factor for MACE in patients referred for SAVR. Of notice, our present adjusted analysis showed that SAVR was not significantly associated with MACE, suggesting that this intervention is protective against mortality but not against risk of adverse cardiovascular events.

Inflammation is a crucial component of atherothrombosis, and elevated levels of hs–CRP has been demonstrated in patients with CAD [17] as well as having an association with increased risk of cardiovascular events [18]. Atherosclerosis and calcific aortic valve disease share similar pathophysiological processes [19] and risk factors, [20] with inflammation being one potential common feature. While elevated hs–CRP levels have been reported in patients with severe AS, [21] hs–CRP is not an established risk factor for progressive valve narrowing [22] and is not clearly associated with outcome [10]. Our present results also showed that hs–CRP was of limited value in terms of predicting mortality or MACE among operated patients.

While most studies of AS have analyzed only one biomarker, here we examined the prognostic impact of three established cardiovascular disease biomarkers. We further examined the combined use of these biomarkers, and our findings suggest that elevated levels of biomarkers was associated with decreased survival, especially among unoperated patients. This finding may implicate that alertness and maybe a re–evaluation of treatment strategy is warranted in patients with elevations of all 3 biomarkers who are initially referred to continued medical treatment. Interestingly, there was no difference in cardiac–specific mortality between groups of elevated biomarkers (ie. “all normal”, “one elevated” or “all 3 elevated”) in operated patients, but cardiac–specific deaths occurred sooner (S3 Fig). This finding may implicate that preoperative biomarker–elevations express the presence of cardiac–risk factors associated with higher short–term mortality and that careful postoperative follow–up and optimization of cardiac–care is warranted. Notably, the present study used reference levels specific to the laboratory at our tertiary center, which are not necessarily specific to AS or even heart disease. However, analysis of NT–proBNP, hsTnT, and hs–CRP is routinely available at most hospitals, and we believe that this feasibility makes our results particularly interesting from a clinical perspective.

For patients with severe AS, the presence of symptoms is still the predominant marker of the transition from watchful waiting to surgical management. Although the assessment of symptoms is straightforward in some patients, evaluation of subjects with asymptomatic severe AS or moderate symptomatic AS remains difficult. Elderly patients often have co–existing comorbidities and may attribute their symptoms to other conditions, thereby disregarding that their reduced exercise capacity could be caused by AS. This may lead to under–reporting the severity of symptoms and preclude them from being considered eligible for AVR. This study does not offer a solution to the aforementioned challenges, but suggest that there might be ways of predicting adverse events (MACE) by use of biomarkers (hsTnT), which in contrast to symptoms, are not subjective measurements. The decision to operate or not is ultimately a question of risk versus benefit, and the risk of adverse outcomes is important to consider. Our findings may suggest that a closer postoperative follow–up in patients with elevated biomarker levels is warranted in order to reduce the incidence of adverse events. Furthermore, our data gives insight into the consequences of remaining unoperated, perhaps promoting a reconsideration in cases where either patients or clinicians are inclined to opt for conservative treatment. By demonstrating that clinically available biomarkers may be used as prognostic markers of adverse outcomes in operated patients, we believe that our data may contribute to the overall risk–assessment and may benefit the discussion of whether or not to undergo SAVR alongside established surgical risk–scoring tools.

## Limitations

The present study has some limitations. Firstly, the single–center design reduces the generalizability of our results. Moreover, patient self–selection likely introduced some bias, as our study included individuals who were motivated to seek a referral to a tertiary center. Secondly, our study was not randomized, which is ethically unfeasible. The groups of operated and unoperated patients had vastly different baseline characteristics. Thirdly, the group of unoperated patients was small and included both very high–risk individuals who were ineligible for intervention, and lower risk individuals who were asymptomatic or who refused intervention. Accordingly, this group of patients was not considered eligible for multivariate analysis. Fourth, we did not include patients referred to TAVI because of a low number of such interventions being conducted at our centre during the study period. Finally, we acknowledge that the risk of false conclusions increase with the number of analyses at our chosen significance level, especially with small numbers of patients in subgroup–analyses.

## Conclusions

In the present cohort of patients with severe AS, we found that hsTnT, as an available, robust, and therefore clinically relevant biomarker was independently associated with the risk of MACE following SAVR. Furthermore, depending on whether the serum levels were below relative to normal values or not, the assessment of NT–proBNP, hsTnT and hs–CRP in combination may have prognostic value in terms of mortality, especially in patients with severe AS referred to continued medical treatment. Overall, our results suggest that all three biomarkers, but particularly hsTnT, can should be further evaluated as prognostic markers in patients with severe AS who are referred to a tertiary center for evaluation of possible aortic valve replacement.

## Supporting information

S1 Fig Kaplan–Meier curves (3–year cardiac–specific death) according to number of biomarkers with preoperative elevated levels (NT–proBNP, hsTnT and / or hs–CRP) for patients referred to undergo surgical aortic valve replacement. (TIF)

S2 Fig Kaplan–Meier curves (3–year cardiac–specific death) according to number of biomarkers with elevated levels (NT–proBNP, hsTnT and / or hs–CRP) at presurgical evaluation for patients referred to continued medical treatment (unoperated patients). (TIF)

S3 Fig Kaplan Meier survival curves (3–year cardiac–specific death) among patients with at least one (or more, including 3 elevated biomarkers) biomarker (NT–proBNP, hsTnT and / or hs–CRP) with elevated levels at presurgical evaluation for patients referred to undergo surgical aortic valve replacement. (TIF)

S4 Fig Kaplan Meier survival curves (3–year cardiac–specific death) among patients with at least one (or more, including 3 elevated biomarkers) biomarker (NT–proBNP, hsTnT and / or hs–CRP) with elevated levels at presurgical evaluation for patients referred to continued medical treatment (unoperated patients). (TIF)

S1 Table Association between enrollment clinical variables and baseline levels of NT–proBNP. NT–proBNP, N–terminal pro–brain natriuretic peptide; NYHA, New York Heart Association; eGFR estimated glomerular filtration rate (Cockroft–Gault formula); hsTnT high sensitive troponin T; hs–CRP, high sensitive C–reactive protein; LVEF, left ventricular ejection fraction; LVCO, left ventricular cardiac output; ACEi, angiotensin converting enzyme; ARB, aldosterone receptor blocker.
(DOCX)

S2 Table Association between enrollment clinical variables and baseline levels of hsTnT. hsTnT, high sensitive troponin T; NYHA, New York Heart Association; eGFR estimated glomerular filtration rate (Cockroft–Gault formula); NT–proBNP, N–terminal pro–brain natriuretic peptide; hs–CRP, high sensitive C–reactive protein; LVEF, left ventricular ejection fraction; LVCO, left ventricular cardiac output; ACEi, angiotensin converting enzyme; ARB, aldosterone receptor blocker.
(DOCX)

S3 Table Association between enrollment clinical variables and baseline levels of hs–CRP. hs–CRP, high sensitive C–reactive protein; NYHA, New York Heart Association; eGFR estimated glomerular filtration rate (Cockroft–Gault formula); NT–proBNP, N–terminal pro–brain natriuretic peptide; hsTnT, high sensitive troponin T; LVEF, left ventricular ejection fraction; LVCO, left ventricular cardiac output; ACEi, angiotensin converting enzyme; ARB, aldosterone receptor blocker.
(DOCX)

S4 Table Univariate–and multivariable analysis for the association of clinical variables with 3–year all–cause mortality or with MACE in operated patients. MACE, major adverse cardiovascular event; HR, hazard ratio; CI, confidence interval; eGFR, estimated glomerular filtration rate; NYHA, New York Heart Association; LVEF. Left ventricular ejection fraction; NT–proBNP, N–terminal pro brain natriuretic peptide; hs–TnT, high–sensitive troponin T hs–CRP, high–sensitive C–reactive protein.
(DOCX)
