# Supplementary figures and images for: Associations of brain–natriuretic peptide, high–sensitive troponin T, and high–sensitive C–reactive protein with outcomes in severe aortic stenosis
Source: PLoS One. 2017 Jun 12;12(6):e0179304. doi: 10.1371/journal.pone.0179304 (PMC5467862; doi:10.1371/journal.pone.0179304)

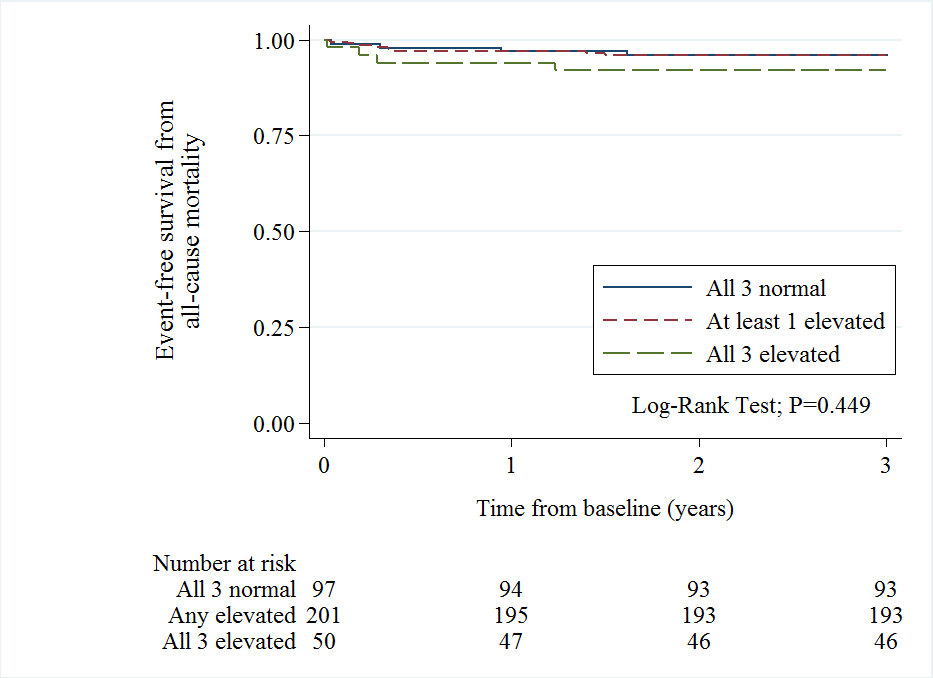

Supplement: S1 Fig — (TIF) [file pone.0179304.s001.tif]

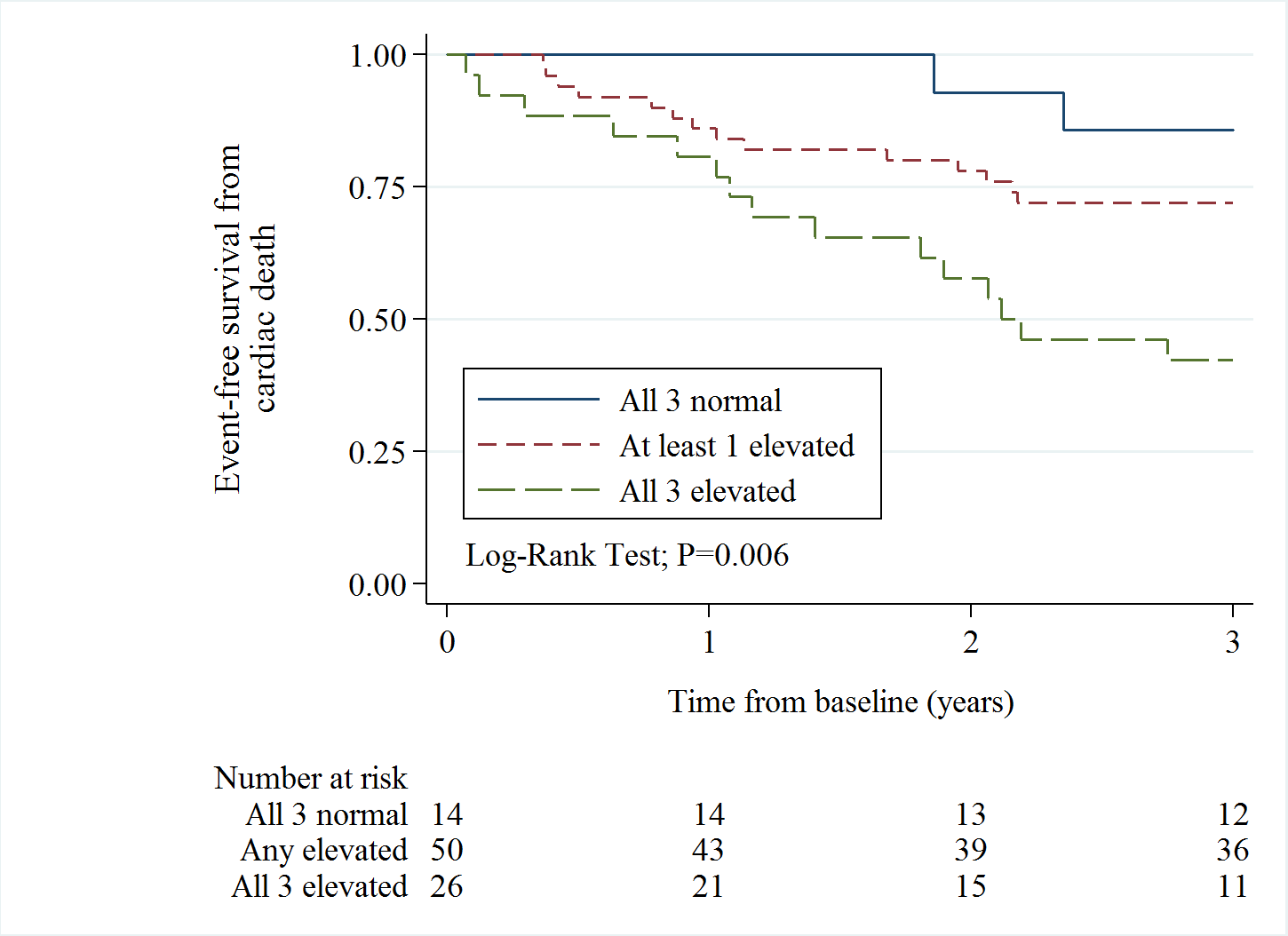

Supplement: S2 Fig — (TIF) [file pone.0179304.s002.tif]

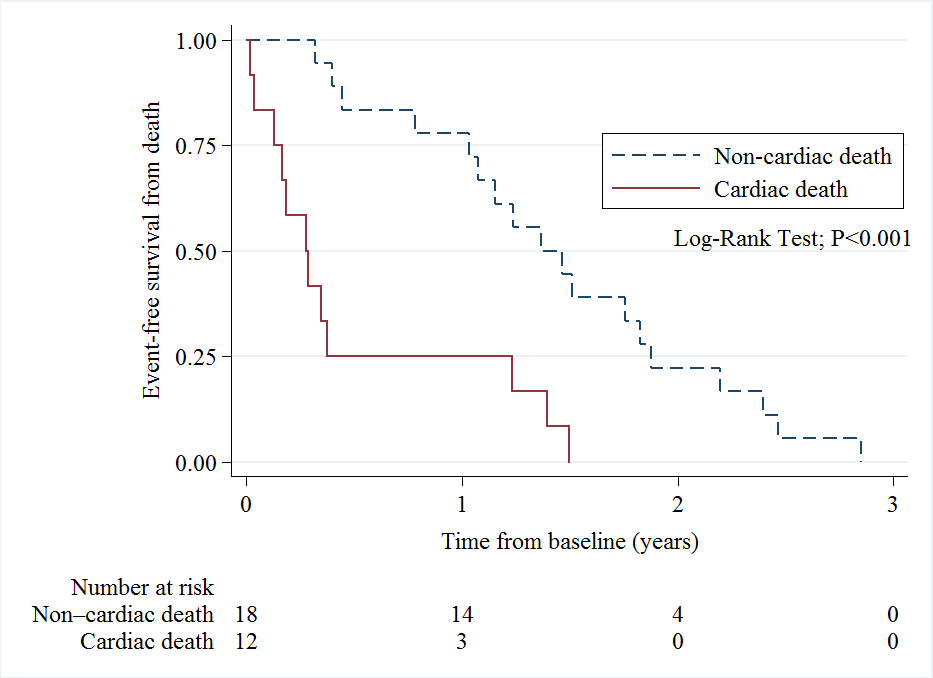

Supplement: S3 Fig — (TIF) [file pone.0179304.s003.tif]

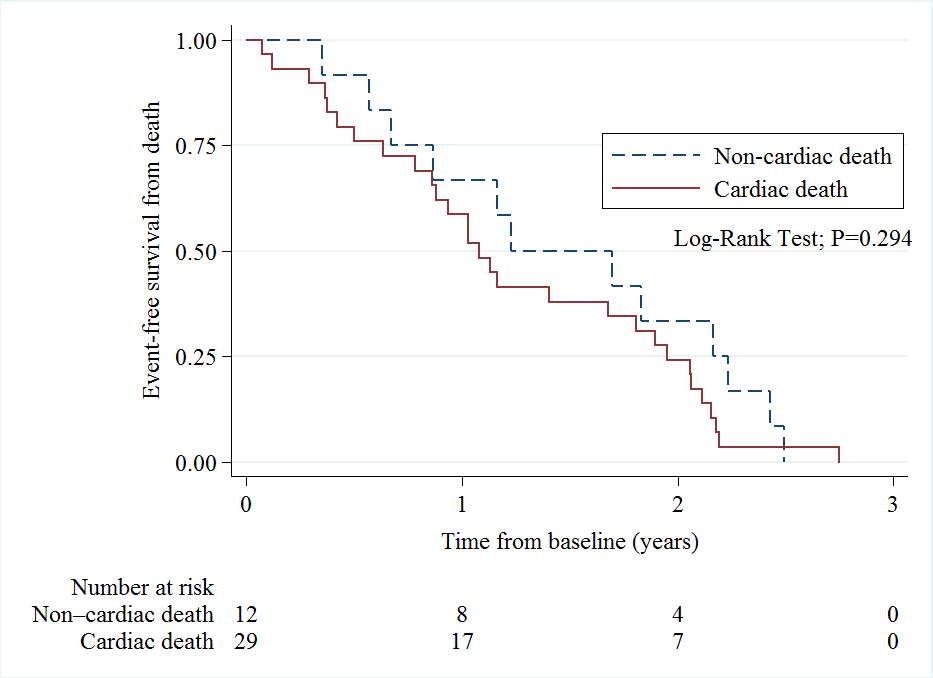

Supplement: S4 Fig — (TIF) [file pone.0179304.s004.tif]
