# Supplementary material for: Associations of brain–natriuretic peptide, high–sensitive troponin T, and high–sensitive C–reactive protein with outcomes in severe aortic stenosis
Source: PLoS One. 2017 Jun 12;12(6):e0179304. doi: 10.1371/journal.pone.0179304 (PMC5467862; doi:10.1371/journal.pone.0179304)
Supplement: S1 Table — NT–proBNP, N–terminal pro–brain natriuretic peptide; NYHA, New York Heart Association; eGFR estimated glomerular filtration rate (Cockroft–Gault formula); hsTnT high sensitive troponin T; hs–CRP, high sensitive C–reactive protein; LVEF, left ventricular ejection fraction; LVCO, left ventricular cardiac output; ACEi, angiotensin converting enzyme; ARB, aldosterone receptor blocker. (DOCX) [file pone.0179304.s005.docx]

Table S1. Association between enrollment clinical variables and baseline levels of NT-proBNP

| **Variables** | **All patients**  **with NT-proBNP**  **(n=432)** | **Tertile 1**  **(<44.5 pmol/L)**  **n=144** | **Tertile 2**  **(44.5-169 pmol/L)**  **n=144** | **Tertile 3**  **(>169 pmol/L)**  **n=144** | ***P*** |
| --- | --- | --- | --- | --- | --- |
| **Demography** |  |  |  |  |  |
| Age, years | 75±11 | 68±12 | 75±9 | 80±8 | <0.001 |
| Male sex – n (%) | 242 (56) | 88 (61) | 78 (54) | 76 (53) | 0.312 |
| Body mass index, kg/m^2^ | 25.9±4.5 | 27.1±4.4 | 26.0±4.4 | 24.6±4.3 | <0.001 |
| NYHA class  I / II / III or IV, % | 11/46/43 | 8/60/32 | 15/47/38 | 10/32/58 | 0.000 |
| Coronary artery disease | 129 (30) | 33 (23) | 42 (29) | 54 (38) | 0.025 |
| Diabetes mellitus | 46 (11) | 11 (8) | 19 (13) | 16 (11) | 0.304 |
| Hypertension | 195 (45) | 61 (42) | 66 (46) | 68 (47) | 0.695 |
| Atrial fibrillation | 92 (21) | 5 (3) | 28 (19) | 59 (41) | 0.000 |
| **Biochemistry** |  |  |  |  |  |
| Creatinine, μmol/L | 87±29 | 78±18 | 84±27 | 99±35 | <0.001 |
| eGFR, ml/min | 74±33 | 92±35 | 73±26 | 56±25 | <0.001 |
| NT-proBNP, median(IQR), pmol/L | 91 (234, 33) | 25 (33, 14) | 91 (123, 60) | 335 (547, 234) | <0.001 |
| hsTnT, ng/L | 13 (25, 10) | 10 (13, 10) | 13 (21, 10) | 26 (42, 15) | <0.001 |
| hs-CRP, mg/L | 2.0 (5.3, 0.8) | 1.4 (3.1, 0.7) | 2.0 (4.8, 0.8) | 3.5 (7.7, 0.9) | 0.024 |
| **Hemodynamics** |  |  |  |  |  |
| LVEF, % | 56±9 | 60±6 | 57±8 | 52±11 | <0.001 |
| LVCO, l/min | 4.9±1.2 | 5.2±1.2 | 5.0±1.2 | 4.4±1.2 | <0.001 |
| Aortic valve area, cm^2^ | 0.7±0.2 | 0.8±0.2 | 0.7±0.2 | 0.6±0.2 | <0.001 |
| Mean aortic gradient, mmHg | 55±18 | 51±15 | 57±17 | 57±21 | 0.005 |
| **Medication** |  |  |  |  |  |
| ACEi or ARB | 172 (40) | 53 (37) | 56 (39) | 63 (44) | 0.464 |
| Beta-blocker | 200 (46) | 34 (24) | 66 (46) | 100 (69) | 0.000 |
| Warfarin | 89 (21) | 7 (5) | 23 (16) | 59 (41) | 0.000 |
| Aspirin | 225 (52) | 74 (51) | 81 (56) | 70 (49) | 0.422 |

NT-proBNP, N-terminal pro-brain natriuretic peptide; NYHA, New York Heart Association; eGFR estimated glomerular filtration rate (Cockroft-Gault formula); hsTnT high sensitive troponin T; hs-CRP, high sensitive C-reactive protein; LVEF, left ventricular ejection fraction; LVCO, left ventricular cardiac output; ACEi, angiotensin converting enzyme; ARB, aldosterone receptor blocker
