# Supplementary material for: Associations of brain–natriuretic peptide, high–sensitive troponin T, and high–sensitive C–reactive protein with outcomes in severe aortic stenosis
Source: PLoS One. 2017 Jun 12;12(6):e0179304. doi: 10.1371/journal.pone.0179304 (PMC5467862; doi:10.1371/journal.pone.0179304)
Supplement: S2 Table — hsTnT, high sensitive troponin T; NYHA, New York Heart Association; eGFR estimated glomerular filtration rate (Cockroft–Gault formula); NT–proBNP, N–terminal pro–brain natriuretic peptide; hs–CRP, high sensitive C–reactive protein; LVEF, left ventricular ejection fraction; LVCO, left ventricular cardiac output; ACEi, angiotensin converting enzyme; ARB, aldosterone receptor blocker. (DOCX) [file pone.0179304.s006.docx]

Table S2. Association between enrollment clinical variables and baseline levels of hsTnT

| **Variables** | **All patients**  **with hsTnT**  **(n=440)** | **Tertile 1**  **(<11 ng/L)**  **n=175** | **Tertile 2**  **(11-21 ng/L)**  **n=124** | **Tertile 3**  **(>21 ng/L)**  **n=141** | ***P*** |
| --- | --- | --- | --- | --- | --- |
| **Demography** |  |  |  |  |  |
| Age, years | 75±11 | 70±11 | 76±9 | 79±9 | <0.001 |
| Male sex – n (%) | 249 (57) | 81 (46) | 74 (60) | 94 (67) | 0.001 |
| Body mass index, kg/m^2^ | 25.9±4.6 | 26.2±4.6 | 25.6±4.9 | 25.9±4.2 | 0.495 |
| NYHA class  I / II / III or IV, % | 11/47/43 | 12/52/36 | 13/46/41 | 8/40/52 | 0.066 |
| Coronary artery disease | 131 (30) | 37 (21) | 36 (29) | 58 (41) | 0.001 |
| Diabetes mellitus | 47 (11) | 13 (7) | 10 (8) | 24 (17) | 0.012 |
| Hypertension | 200 (45) | 69 (39) | 60 (48) | 71 (50) | 0.113 |
| Atrial fibrillation | 93 (21) | 22 (13) | 28 (23) | 43 (31) | <0.001 |
| **Biochemistry** |  |  |  |  |  |
| Creatinine, μmol/L | 87±29 | 75±17 | 85±22 | 103±38 | <0.001 |
| eGFR, ml/min | 74±33 | 86±33 | 70±29 | 62±30 | <0.001 |
| NT-proBNP, median(IQR), pmol/L | 90 (234, 33) | 38 (91, 18) | 80 (187, 36) | 241 (470, 124) | <0.001 |
| hsTnT, ng/L | 13 (25, 10) | 10 (10, 10) | 14 (17, 12) | 35 (45, 25) | <0.001 |
| hs-CRP, mg/L | 2.0 (5.3, 0.8) | 1.3 (3.9, 0.6) | 2.0 (4.8, 0.9) | 3.5 (8.9, 1.2) | <0.001 |
| **Hemodynamics** |  |  |  |  |  |
| LVEF, % | 56±9 | 59±7 | 56±9 | 53±11 | <0.001 |
| LVCO, l/min | 4.9±1.2 | 5.1±1.2 | 4.7±1.1 | 4.7±1.3 | 0.004 |
| Aortic valve area, cm^2^ | 0.7±0.2 | 0.7±0.2 | 0.7±0.2 | 0.6±0.2 | <0.001 |
| Mean aortic gradient, mmHg | 55±18 | 54±16 | 54±18 | 57±20 | 0.155 |
| **Medication** |  |  |  |  |  |
| ACEi or ARB | 176 (40) | 65 (36) | 49 (40) | 62 (44) | 0.524 |
| Beta-blocker | 205 (47) | 60 (34) | 61 (49) | 84 (60) | <0.001 |
| Warfarin | 89 (20) | 7 (5) | 25 (20) | 23 (13) | 0.002 |
| Aspirin | 231 (53) | 70 (56) | 70 (56) | 78 (55) | 0.220 |

hsTnT, high sensitive troponin T; NYHA, New York Heart Association; eGFR estimated glomerular filtration rate (Cockroft-Gault formula); NT-proBNP, N-terminal pro-brain natriuretic peptide; hs-CRP, high sensitive C-reactive protein; LVEF, left ventricular ejection fraction; LVCO, left ventricular cardiac output; ACEi, angiotensin converting enzyme; ARB, aldosterone receptor blocker
