# Supplementary material for: Associations of brain–natriuretic peptide, high–sensitive troponin T, and high–sensitive C–reactive protein with outcomes in severe aortic stenosis
Source: PLoS One. 2017 Jun 12;12(6):e0179304. doi: 10.1371/journal.pone.0179304 (PMC5467862; doi:10.1371/journal.pone.0179304)
Supplement: S3 Table — hs–CRP, high sensitive C–reactive protein; NYHA, New York Heart Association; eGFR estimated glomerular filtration rate (Cockroft–Gault formula); NT–proBNP, N–terminal pro–brain natriuretic peptide; hsTnT, high sensitive troponin T; LVEF, left ventricular ejection fraction; LVCO, left ventricular cardiac output; ACEi, angiotensin converting enzyme; ARB, aldosterone receptor blocker. (DOCX) [file pone.0179304.s007.docx]

Table S3. Association between enrollment clinical variables and baseline levels of hs-CRP

| **Variables** | **All patients**  **with hs-CRP**  **(n=438)** | **Tertile 1**  **(<0.6 mg/L)**  **n=142** | **Tertile 2**  **(0.6-3.9 mg/L)**  **n=147** | **Tertile 3**  **(>3.9 mg/L)**  **n=149** | ***P*** |
| --- | --- | --- | --- | --- | --- |
| **Demography** |  |  |  |  |  |
| Age, years | 74±11 | 74±12 | 74±10 | 75±10 | 0.972 |
| Male sex – n (%) | 246 (56) | 68 (48) | 93 (63) | 85 (57) | 0.030 |
| Body mass index, kg/m^2^ | 26.0±4.6 | 24.8±4.0 | 26.7±4.4 | 26.3±5.0 | 0.001 |
| NYHA class  I / II / III or IV, % | 11/46/43 | 14/49/37 | 9/53/38 | 9/38/53 | 0.017 |
| Coronary artery disease | 130 (30) | 39 (27) | 36 (24) | 55 (37) | 0.051 |
| Diabetes mellitus | 47 (11) | 15 (11) | 18 (12) | 14 (9) | 0.729 |
| Hypertension | 199 (45) | 62 (44) | 68 (46) | 69 (46) | 0.875 |
| Atrial fibrillation | 93 (21) | 22 (15) | 28 (19) | 43 (29) | 0.015 |
| **Biochemistry** |  |  |  |  |  |
| Creatinine, μmol/L | 87±29 | 78±19 | 88±27 | 94±36 | <0.001 |
| eGFR, ml/min | 74±33 | 73±29 | 77±35 | 72±33 | 0.330 |
| NT-proBNP, median(IQR), pmol/L | 88 (237, 33) | 62 (180, 29) | 65 (172, 31) | 120 (174, 109) | 0.001 |
| hsTnT, ng/L | 13 (25, 10) | 10 (19, 10) | 13 (24, 10) | 20 (28, 10) | 0.001 |
| hs-CRP, mg/L | 2.0 (5.3, 0.8) | 0.6 (0.8, 0.6) | 1.9 (2.6, 1.4) | 7.7 (13, 5.3) | <0.001 |
| **Hemodynamics** |  |  |  |  |  |
| LVEF, % | 56±9 | 57±8 | 57±8 | 54±11 | 0.007 |
| LVCO, l/min | 4.9±1.2 | 4.9±1.2 | 4.9±1.3 | 4.8±1.3 | 0.734 |
| Aortic valve area, cm^2^ | 0.7±0.2 | 0.7±0.2 | 0.7±0.2 | 0.7±0.2 | 0.436 |
| Mean aortic gradient, mmHg | 55±18 | 57±18 | 55±17 | 53±19 | 0.327 |
| **Medication** |  |  |  |  |  |
| ACEi or ARB | 176 (40) | 61 (43) | 52 (35) | 63 (42) | 0.407 |
| Beta-blocker | 204 (47) | 56 (39) | 63 (43) | 85 (57) | 0.006 |
| Warfarin | 89 (20) | 19 (13) | 26 (18) | 44 (30) | 0.002 |
| Aspirin | 230 (53) | 86 (61) | 79 (54) | 65 (44) | 0.014 |

hs-CRP, high sensitive C-reactive protein; NYHA, New York Heart Association; eGFR estimated glomerular filtration rate (Cockroft-Gault formula); NT-proBNP, N-terminal pro-brain natriuretic peptide; hsTnT, high sensitive troponin T; LVEF, left ventricular ejection fraction; LVCO, left ventricular cardiac output; ACEi, angiotensin converting enzyme; ARB, aldosterone receptor blocker
