# Supplementary material for: Associations of brain–natriuretic peptide, high–sensitive troponin T, and high–sensitive C–reactive protein with outcomes in severe aortic stenosis
Source: PLoS One. 2017 Jun 12;12(6):e0179304. doi: 10.1371/journal.pone.0179304 (PMC5467862; doi:10.1371/journal.pone.0179304)
Supplement: S4 Table — MACE, major adverse cardiovascular event; HR, hazard ratio; CI, confidence interval; eGFR, estimated glomerular filtration rate; NYHA, New York Heart Association; LVEF. Left ventricular ejection fraction; NT–proBNP, N–terminal pro brain natriuretic peptide; hs–TnT, high–sensitive troponin T hs–CRP, high–sensitive C–reactive protein. (DOCX) [file pone.0179304.s008.docx]

|  | **3–YEAR MORTALITY** | | | | **MACE** | | | |
| --- | --- | --- | --- | --- | --- | --- | --- | --- |
|  | Univariate | | Multivariate | | Univariate | | Multivariate | |
|  | HR (95% CI) | P | HR (95% CI) | P | HR (95% CI) | P | HR (95% CI) | P |
| Age (decade) | 1.75 (1.17–2.62) | 0.006 | 1.24 (0.70–2.21) | 0.456 | 1.01 (0.98–1.04) | 0.537 | 0.87 (0.56–1.35) | 0.544 |
| Gender, male | 1.08 (0.55–2.12) | 0.828 | 1.37 (0.63–2.96) | 0.427 | 1.75 (0.91–3.34) | 0.092 | 1.38 (0.68–2.80) | 0.377 |
| [Log_e_] eGFR | 0.58 (0.41–0.83) | 0.003 | 0.62 (0.36–1.08) | 0.093 | 0.55 (0.11–2.91) | 0.483 | 0.89 (0.55–1.41) | 0.605 |
| NYHA > 2 | 2.28 (1.15–4.53) | 0.018 | 1.99 (0.94–4.22) | 0.074 | 1.71 (0.94–3.11) | 0.077 | 1.81 (0.96–3.43) | 0.067 |
| LVEF | 0.96 (0.93–1.00) | 0.030 | 0.97 (0.94–1.00) | 0.074 | 0.96 (0.93–0.98) | 0.002 | 0.97 (0.94–0.99) | 0.043 |
| [Log_e_] NT–proBNP | 1.86 (1.27–2.72) | 0.001 |  |  | 1.32 (0.96–1.81) | 0.092 |  |  |
| [Log_e_] hsTnT | 1.24 (0.95–1.62) | 0.106 |  |  | 1.40 (1.12–1.75) | 0.003 | 1.51 (1.11–2.05) | 0.008 |
| [Log_e_] hs–CRP | 1.14 (0.81–1.59) | 0.450 |  |  | 1.11 (0.82–1.51) | 0.493 |  |  |

MACE, major adverse cardiovascular event; HR, hazard ratio; CI, confidence interval; eGFR, estimated glomerular filtration rate; NYHA, New York Heart Association; LVEF. Left ventricular ejection fraction; NT–proBNP, N–terminal pro brain natriuretic peptide; hs–TnT, high–sensitive troponin T hs–CRP, high–sensitive C–reactive protein.
